# Heavy Metals in Cannabis Vapes and Their Health Implications—A Scoping Review

**DOI:** 10.1155/tswj/9529544

**Published:** 2025-08-19

**Authors:** Sumit Gaur, Rupali Agnihotri

**Affiliations:** ^1^Department of Pedodontics and Preventive Dentistry, Manipal College of Dental Sciences, Manipal, Manipal Academy of Higher Education, Manipal, Karnataka, India; ^2^Department of Periodontology, Manipal College of Dental Sciences, Manipal, Manipal Academy of Higher Education, Manipal, Karnataka, India

**Keywords:** cannabis electronic cigarette, cannabis vaping, health effects, metals

## Abstract

Cannabis vaping involves the vaporization of cannabis vape (CV) liquid via a vape pen made of metallic and nonmetallic parts. Recently, various metal contaminants that originate from cannabis, as well as the vape liquid packaging device, were reported in CV liquids and aerosols. Heavy metal exposure from CVs is associated with various health afflictions and should be regulated. This scoping review intends to investigate the literature on heavy metal releases from CVs and their impact on health. This review was conducted according to the PRISMA-ScR scoping review guidelines. Initial search in electronic databases such as Medline (PubMed), Scopus, Embase, and Web of Science yielded nine studies published until February 2024. The metals released from CVs, the variables influencing their leaching, and any documented adverse health impacts were recorded. Findings revealed that the structural elements of the CVs leached metals such as nickel (Ni), chromium (Cr), lead (Pb), cobalt (Co), cadmium (Cd), and copper (Cu), which were transported into the aerosol as tiny particles. Several factors, including the structural integrity of the device components, device age, operating temperature, vape liquid pH, and viscosity cutting agents in the vape liquid, caused metal dissolution. Even though none of the studies evaluated the direct health impact of these metals, they proposed that they may affect multiple body organs, specifically the lungs, where they were associated with electronic vaping device use-associated lung injury (EVALI). These metals might expedite the transformation of vitamin E acetate into ketenes, which are associated with EVALI. We can conclude that heavy metals beyond the regulatory guidelines are released from CVs and have negative health consequences. Further research is required to improve the CV design elements to lower the metal leaching. Additionally, disclosing the CV packaging contents to consumers is essential to prevent possible health risks.

## 1. Introduction

The consumption of tobacco and cannabis constitutes significant public health concerns. Although the health hazards of toxicants from conventional cigarettes are well documented, there is scarce information about the health implications of the so-called safer alternatives to cigarettes, electronic cigarettes (ECs), or vapes. The ECs or vapes are very popular among youngsters in the United States between 18 and 24 years of age [1]. A wide range of cannabis products entered the markets when it was legalized in both Canada and the United States [2]. Traditionally, cannabis was used by burning the “flower/bud” of the plant, which was lit, smoked, and inhaled through a smoking device [3]. However, currently, vaping is the most common method of cannabis and nicotine consumption [4, 5]. In the United States, the percentage of college students exposed to cannabis vapes (CVs) in their lifetime ranged from 9% to 29%, while the same rate in high school students was between 5% and 9% [6–9].

Conventional cannabis vaporizers heat dried cannabis herb or the liquid cannabis extracts to high temperatures, leading to cannabinoid vaporization [10]. Tetrahydrocannabinol (THC), the active component of cannabis, is solubilized in an e-liquid formulation, including propylene glycol or vegetable glycerin, occasionally enhanced with flavorings [11]. Vaping cannabis liquid is a noncombustion method in which the concentrate is breathed through a mouthpiece after being aerosolized when it comes into contact with a resistance-heated element [12]. Due to the variation in device design and e-liquid composition, concerns remain over the toxicants present, the health hazards to the airways, and the physiological responses elicited in users. It is thought that EC aerosols have fewer toxic compounds than regular tobacco and, possibly, marijuana cigarettes [13]. It is challenging to research the differences in their emission products because the use of cannabis and nicotine ECs differs from that of tobacco cigarettes. In addition to psychotropic compounds like nicotine and cannabinoids (primarily THC and CBD), the aerosols released from vaping products also contain respiratory toxicants such as formaldehyde, acrolein, and benzaldehyde [14].

The vape liquid and aerosol of nicotine ECs were shown to have higher levels of harmful metals such as nickel (Ni), chromium (Cr), and lead (Pb) [15]. Because of corrosion or surface cracking, metallic parts of vaping devices can leach Pb, Ni, copper (Cu), zinc (Zn), tin (Sn), aluminum (Al), and Cr into the vape liquid [16]. The aerosol and vape liquid in the cartridge tank contained more metals than the vape liquid in the dispenser, indicating that they migrated from the coil [17]. Owing to their small nanoparticle size (< 100 nm), these metals can be easily inhaled by the consumers from the aerosol [16]. Besides the device component, the *Cannabis sativa* plant can be a metal source as it is a hyperaccumulator of metals which quickly absorbs them from the contaminated soil [18]. Its leaves, roots, and stems have high concentrations of Cu, cadmium (Cd), Ni, and Cr [18, 19]. These naturally accumulated metals in cannabis may not entirely be removed through extraction and may be present in the final products derived from the plant [20]. Although heavy metals were detected in tobacco and cannabis under various smoking conditions, very few investigations report their presence in the CVs [12, 21]. Like the nicotine ECs, the heavy metals might leak from the structural elements of CVs into the concentrate depending on the temperature, oil acidity, frequency, and duration of usage [22]. Simultaneous use of CVs and nicotine may result in prolonged exposure to dangerous metals, which may be detrimental to the adult's and child's health [23].

Even though many chemical components of vaping, nicotine, and cannabinoids are similar, some are distinct and significant when considering the respiratory consequences of vaping both substances [24]. For instance, cannabinoids are lipophilic, so solvents used in CV liquids differ from nicotine ECs [25]. Aerosols of nicotine and CVs also contain hydrophobic chemicals such as vitamin E acetate (VEA), which were found in bronchoalveolar lavage fluid and were associated with increased risk of electronic cigarette or vaping device use-associated lung injury (EVALI) [23, 26].

Evidence suggests that the body reacts to vape aerosol similar to that of cigarette smoke [27]. Just like cigarette smoke, exposure to e-liquid caused similar degrees of cellular damage and morbidity in cultures of human skin and lung cells [28]. In a mouse model, vape liquid increased the synthesis of various proinflammatory cytokines. Its effects on lung function were comparable to cigarette smoke [29]. Studies on animal inhalation and in vitro cytotoxicity also demonstrated more oxidative stress, lung damage, and inflammatory alterations upon vape aerosol inhalation than nicotine [30]. It caused a stronger inflammatory response and pathological alterations linked to lung damage. Lastly, heavy metals like Pb in CV liquids, although lower than those in cigarette smoke, may produce adverse health effects [31].

Unlike the nicotine ECs, CV aerosols lack a thorough characterization of hazardous metals [21]. Considering increased cannabis vaping among teenagers and young adults, it is critical to analyze these potentially hazardous materials, like toxic metals in CVs, their concentrates, and aerosols, which may negatively impact multiple body organs, including the lungs [23]. Given the above, this scoping review was aimed at comprehensively exploring and mapping the literature on the heavy metals released from CVs and their health implications.

## 2. Materials and Methods

### 2.1. Research Question

The specific research question was as follows: what are the heavy metals released from CVs, and what are their health impacts? This specific research question would enable the exploration of harmful metals released from CVs, factors that affect their release, and their harmful effects on the health of youngsters and adults using them.

### 2.2. Inclusion Criteria

This scoping review included all study designs assessing heavy metals released from CVs and their adverse health implications. The inclusion criteria were full texts of all original peer-reviewed studies until February 20, 2024, about the occurrence of heavy metals in CVs and their potential health effects.

### 2.3. Exclusion Criteria

Any reviews, abstracts, conference proceedings, letters, commentaries, opinions, and book chapters were excluded. Additionally, articles published in languages other than English were excluded.

### 2.4. Search Strategy

**Figure 1:**
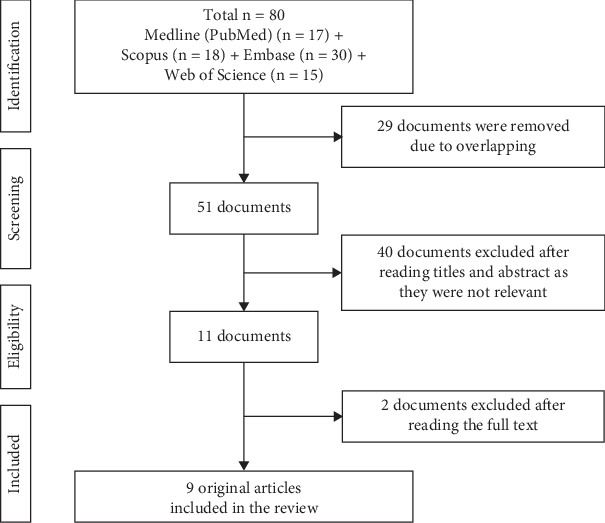
Evidence search for the heavy metals in cannabis vapes and their health implications.

The research publications pertaining to the occurrence of heavy metals in CVs and their potential health effects were sought out through a search of various online databases, such as Medline (PubMed), Scopus, Embase, and Web of Science. Specific search methods were developed for every database. All fields were searched using a combination of keywords such as “Cannabis” AND “Vaporizer” OR “Electronic Cigarette” OR “Vaping” OR “ENDS” OR “Vape” AND “metals.” There were no year limits. The research selection was strictly based on the PRISMA extension for scoping reviews (PRISMA-scR), as shown in the flow diagram [32] (Figure 1).

### 2.5. Study Selection

The titles and abstracts of the studies were initially reviewed independently by the two reviewers, followed by a thorough evaluation of the full texts. Any disagreements about study inclusion were resolved through discussions.

### 2.6. Data Extraction

A standardized form was used by the two researchers to extract the data independently. Any conflicts were resolved through discussions. The following information was extracted: the last name of the first author, the year the study was published, the component of the CV that was analyzed, the metal released, the method used for its detection, the metal concentration, the factor causing its release, and any possible health implications that were mentioned.

### 2.7. Search Results

Out of the 80 papers found by the electronic search (17 in PubMed, 30 in Embase, 18 in Scopus, and 15 in Web of Science), 51 were present after eliminating duplicates. Following a review of the abstracts and titles by two reviewers, 40 papers were excluded, as they were irrelevant. Lastly, the entire texts of nine original experimental studies were included in the review [12, 21–23, 26, 33–36].

## 3. Results and Discussion

### 3.1. Type of CVs and Their Components Evaluated

The included studies tested commercial, model, and counterfeit CVs to evaluate the presence of metals in CV liquids, aerosols, and structural components. Aerosols produced by the glass tank cannabis delivery system, which mainly used pod-type devices, were examined [22, 23]. It included a 1-g glass tank (MG210, Mr. Green Supply) and a “model” system cartridge with a “510 thread” style [22]. A commercial, nonportable tabletop vaporizer, Volcano Digit, was also assessed [36]. Additionally, a few studies used nicotine and CV cartridges that were taken from EVALI patients [35]. They examined the amount of metal in CV liquids that were either legal or counterfeit [12, 26]. To create aerosols, the oil, flower, and cannabis concentrate were utilized [21, 22]. The metals released from aerosols of CVs and nicotine ECs and their structural components were compared. A study also assessed botanical raw materials (BRMs), which are the ground plant parts (leaves and stems) that are smoked as unfiltered cannabis cigarettes, except the main stem [36]. Additionally, commercially available cartridges with ceramic, plastic, or metallic mouthpieces were examined [22, 35].

### 3.2. Method of Aerosol Generation and Collection

CV aerosol was produced in the experiments with the help of the CETI-8 EC vape [23]. It was collected in a tubing system attached to the vaping machine syringe pump at one end and the mouthpiece of the CVs at the other [23]. In two trials, the CORESTA aerosol collection method with 81 parameters was applied (3 s 55 mL puff every 30 s with a rectangular puff profile) [21, 23]. Aerosol was produced for every sample ranging from 15 [23] to 50 puffs [21, 22], with an approximate 87% recovery in the condensation tubing. One study used a smoke machine configured to a square puff profile with 3 s puffs and 42 s rest between each puff for aerosol generation and collection [22]. In the cannabis flower combustion studies, 200 mg of cannabis flower was ignited in a glass apparatus. With a constant flow rate of 12 mL/s, the combustion smoke and dab aerosols were pulled through the impinger, and the aerosol collected was analyzed for metals after rinsing with acetone [22].

### 3.3. Method Used for Trace Metal Detection in CV E-Liquids

For metals analysis, 0.2–1.0 g of cannabis e-liquid from CVs used by individuals experiencing acute respiratory symptoms associated with EVALI was extracted [33]. As they flow freely at 110°C–120°C, the samples were heated in a microcentrifuge tube to a maximum of 120°C and stirred for 30–60 min before analysis [33]. The studies used inductively coupled plasma mass spectrometry (LA-ICP-MS), ICP-MS, scanning electron microscopy (SEM) with energy-dispersive x-ray spectroscopy (EDS), and portable x-ray fluorescence (XRF) for metal detection. Among these techniques, the ICP-MS was most frequently applied for detecting metals in CV-liquids. It was preferred as it could detect a wide range of metals, including heavy metals, which are of particular concern in CV liquids. Moreover, its high sensitivity and ability to quantify the metals increased its acceptability as an effective method for trace metal detection in CV liquids [37]. The metal particle size varied from hundreds to tens of nanometers [12].

### 3.4. Metals in CV Liquids

The concentrations of various metals in CV liquids are mentioned in Table 1. Four studies reported the metal concentrations in micrograms per gram in the CV liquids [12, 21, 22, 33]. However, their concentrations varied greatly due to differences in the CV liquid samples and estimation methods. Accordingly, the concentration of Pb ranged from 1.2 to 50 *μ*g/g, Ni from 6.2 to 677 *μ*g/g, Cu from 6.4 to 485 *μ*g/g, Cr from 1.5 to 8.02 *μ*g/g, Cd from 0.8 to 7.57 *μ*g/g, Co from 0.968 to 7.99 *μ*g/g, Hg from 0.4 to 3.44 *μ*g/g, and As from 2.0 to 7.33 *μ*g/g. The studies evaluated CV liquids from different sources like illegal vapes [12, 26], EVALI response cartridges [33], commercial vapes [21, 22], and model system vapes [22]. High concentrations of Pb, Ni [12, 26], and Zn were detected in illegal samples of CV liquids, whereas the legal samples contained Cu [12]. The levels of Pb and Ni exceeded the specified tolerance limits by 100 and 900 times, respectively. Co, Cr, manganese (Mn), vanadium (V), and sodium (Na) were among the other elements [12]. The CV liquids from the 2019 EVALI response patients' devices included metals like Cu, Cr, Ni, and Pb (600 ppm) [33]. Additionally, oil from 1-g glass tanks and commercial cartridges with the “510 thread” type were examined before and after vaping [21, 22]. In CV liquids from commercial cartridges, higher levels of Cr, Cu, Ni, and Mn were found before vaping [22]. Similarly, in the model system vape liquids, Cr, Cu, Hg, Mn, and Ni were noted. Additionally, when model CV liquids were heated to 25°C, at 3 weeks, Cu, Hg, Mn, and Ni increased, while at 7 months, additional metals like Co, Cr, Pb, and Sn leached into it. At the same time, the concentration of Cu and Ni significantly rose [22]. Similarly, when heated to 42°C, Cu, Hg, Mn, and Ni were found at 3 weeks, and at 7 months, additional metals like Cd, Cr, Pb, and Sn were identified. Furthermore, Cu, Ni, and Pb levels were abnormally high at 42°C [22]. After vaping, higher quantities of metals like Ni, Cu, Sn, Co, Cr, As, Cd, Pb, and Hg were found in the CV liquid, with Ni and Cu being the highest [21]. Comparing metals in cannabis and nicotine vape liquids showed that Co, Ni, Cu, Zn, Sn, and Pb were the most common metals in CV liquids, whereas Mn, Co, Ni, Cu, Zn, Sn, and Pb were found in nicotine vape liquids [33].

**Figure 2:**
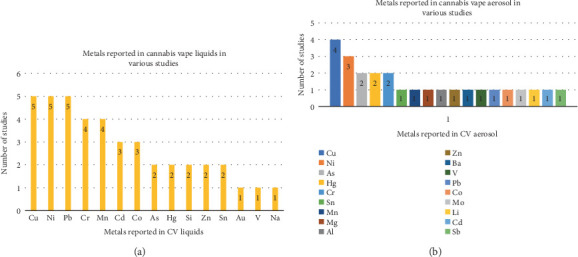
Heavy metals reported in cannabis vape (a) liquids and (b) aerosols.

It was suggested that a lack of good manufacturing practices during extraction may lead to contamination of CV liquids with metals from various processes and/or equipment used for extraction. Some states (e.g., California, Missouri, Colorado, Maine, and Michigan) in the United States have regulatory guidelines for inhaled medical cannabis products that require testing for Cd, Pb, As, and Hg with limits of 0.2, 0.5, 0.2, and 0.1 *μ*g/g, respectively. These limits are derived from the International Council for Harmonization (ICH) Guideline for Elemental Impurities and are based on using 10 g of the material per day as per ICH Option 1. However, in two studies, no products had As, Cd, or Hg concentrations above the limits, but Pb levels were greater than the 0.5 *μ*g/g limit in 21 e-liquid samples in one study [33] and in one legal (0.628 *μ*g/g) and six illegal (range 1.52–48.9 *μ*g/) samples in another study [12]. However, the studies also reported that regulatory limits were related to long-term exposure at waste sites, and a similar model system cannot be applied to CV exposures. Besides, these regulatory guidelines would fail to detect most metals, including the “big four” generally required for cannabis products [22]. Overall, Cu, Ni, Pb, Cd, and Co were most often found metals in the CV liquids [12, 21, 22, 26, 33], and they most likely leached from the metal parts of the CVs, which will be covered in more detail later (Figure 2a).

### 3.5. Metals in CV Aerosol

The aerosol contained metal particles with nanosizes ranging from 20 to 300 nm [12]. High Cu, As, Cr, Hg, Ni, and Sn concentrations were recovered from CV aerosols [21, 23]. The aerosols of both terpenated and unterpenated concentrates included Cu, Cr, Ni, and Mn [22]. In contrast to terpenated aerosol, Pb and Sn were only found in the unterpenated concentrates, which also had higher concentrations of Cr, Cu, Mn, Ni, Pb, and Sn. Additionally, “dabbing” vape liquid raised the aerosol's levels of Cr and Ni, but the smoke from burning flowers revealed higher levels of Mn, Sn, Cu, Cd, and Pb [21, 22]. Elevated Cu, Sn, Ni, and Pb concentrations were also recovered from nicotine ECs in one investigation [22] (Figure 2b).

### 3.6. Metals Leached From the Structural Components of CVs

**Figure 3:**
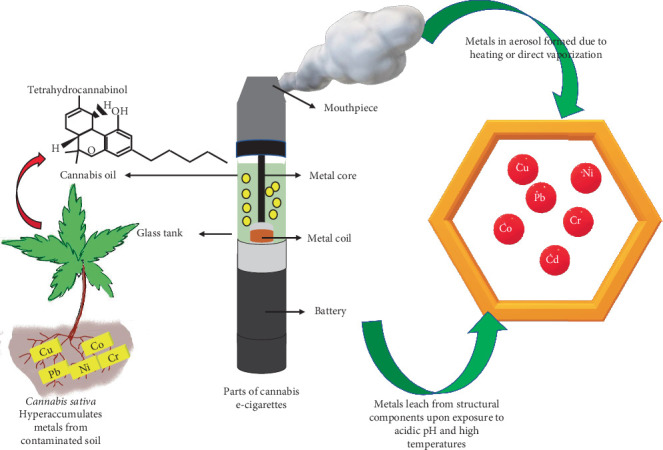
Parts of cannabis vapes and heavy metals released.

Metals like Ni, Cu, Cr, Mg, Zn, Hg, Pb, and Si leached from the vaporizer components [34] (Figure 3). The following different metals were found in the structural elements of CVs.

#### 3.6.1. Heating Element

The heating elements of cannabis cartridges either have a fiberglass and wick heating mechanism or a ceramic heating element, while the nicotine ECs have wick heating elements and fiberglass [22]. The wire from the CV of an EVALI patient showed Ni, Fe, Cr, Co, and S, while the nicotine EC filament had Cr and Ni. Each wick's fiberglass fibrils showed Si and O with trace amounts of Al or Ca [35]. Al and Si comprised most of the ceramic heating block, with Na on the surface [23]. High concentrations of Ni, Cr, Cu, and Pb were found in the heating coil and metal core of CV that was in contact with the chamber's oil. Compared to the model cartridge, the heating coils from the commercially obtained CV cartridges had higher levels of Ni and Cu [22]. The nicotine devices produced Ni and Cr with minimal Fe and zirconium (Zr), whereas the CV filaments yielded predominantly Ni [35]. A nicotine EC's wick, filament, and battery contact showed Fe and Au; their wire leads were mainly made of Ni and brazed to the filaments.

#### 3.6.2. Battery Contact

The predominant metals in the battery contacts of the CV cartridges were Ni [23, 35] and Cr [23]. Besides, minimal Co, Cu, Ni, Zn, Pb, Cr, Fe, Sn, and Zr were also detected. In nicotine ECs, the battery contact gaskets comprised Si, O, C, Ca, Ti, and Zn, while Ni, Fe, and Au were observed on the surface. Moreover, Cu, Ni, and Zn were detected with low quantities of Pb, Sn, and Fe in the coil head body of another commercial nicotine EC [35].

#### 3.6.3. Mouthpiece

Fe, Cr, and Ni were found in the mouthpieces of the CV cartridges. Au was also detected in the nicotine EC cartridges [35]. The commercial cartridges with metal mouthpieces had Cu, Mn, Ni, Pb, and Sn, significantly greater than the ceramic and plastic mouthpieces [22]. The mouthpieces of the model cartridges had Cr and Ni.

The heavy metals present in CV liquids, aerosols, and device components are summarized in Table 1.

### 3.7. Factors Responsible for Increased Release of Metals From CVs

Metals can enter the CV aerosols and liquids through two possible pathways: direct vaporization or leaching of the cartridge components into the oil and transportation by oil droplets or aerosol mixture [12]. The following factors influenced the release of metals from CVs.

#### 3.7.1. Device Age and Storage

The composition of the cannabis extract might change after packaging, during transportation, or storage, as was reported in a CV device approximately 8 months after packaging [12]. The concentration of metals like Zn and Cu differed between the legal CV liquids made from the same lot of cannabis, indicating that the heterogeneity of the extract or changes during storage affected the composition of the vape liquid [12]. Extended storage oxidizes the liquid particles and corrodes the metallic parts, increasing metal concentration. Subsequently, some untested metals might seep from the cartridge components into the CV concentrates under ambient storage settings between filling and purchase and with the age of the cartridge [22].

#### 3.7.2. Composition of the Vape Liquid and Its pH

Cannabis oil has a low pH and relatively high acidity, so long-term contact between the metal parts of the CV and the acidic liquid may cause metal dissolution [12, 22]. Furthermore, viscosity-modifying agents like propylene glycol, unflavored terpenes, and medium-chain triglycerides (e.g., coconut oil) are added to reduce the viscosity of CV liquids. Terpenes, at 5%–15% concentration, act as thinning agents, add flavor, and influence the vaporization of CV liquids and metal migration into the aerosol, as was observed in the commercial vape liquids wherein the terpenes reduced the viscosity and metal content in the vapors [22]. The concentration of metals was higher in unterpenated than the terpenated CV liquids, which may be explained by the “viscosity-only” and “heat of vaporization” theories [22]. According to the “viscosity-only” theory, a low-viscosity oil coats the heating coil faster and more evenly, preventing localized air pockets with higher temperatures. Contrarily, in unterpenated CV liquids, larger air bubbles develop inside the cartridges during vapor collection trials. These air bubbles create a honeycomb appearance, as they cannot escape. The high viscosity of the liquid and lower heat capacity of air further increases the metal levels in the vapor. As the terpenated matrix is less dense, it enhances the oil coating and evaporates more liquid. According to the molecule-specific “heat of vaporization” theory, the smaller terpene molecules volatilize more easily than the bigger cannabis molecules, which have increased heat of vaporization. Subsequently, the heating coil's local temperature drops due to evaporative cooling, stabilizing the metals in the terpenated liquid and preventing their evaporation [22].

#### 3.7.3. Device Temperature

Following vaping, there was a modest increase in the content of heavy metals in commercial CV liquids. The typical temperature range for electronic equipment containing liquids is 135°C–334°C, while dry heating coils can reach up to 1000°C. The vaporization of CV liquids occurred at a high temperature of 200°C–350°C, which reached as high as 600°C in a fully loaded tank, increasing the metal dissolution and liquid contamination similar to nicotine ECs [12, 17]. When the vape liquid levels were low, the heated coil further volatilized the dissolved metals or metal particles from the weakened metal component of CVs [12]. Moreover, heating the liquids to higher temperatures for an extended period resulted in more leaching of the metals from the cartridge devices [22]. Subsequently, identical CVs stored for 3 weeks and 7 months at 25°C and 42°C, to simulate room or elevated temperature, respectively, showed As, Cd, Co, Pb, and Sn with significant amounts of Cu and Ni at 25°C and even higher levels of Cu, Ni, Pb, Sn, Cr, and Mn at 42°C at 7 months. These levels were significantly higher than those found at 3 weeks for both temperatures, indicating that elevated levels of Cu, Cr, Ni, and Pb were directly proportional to the duration of storage and coil temperature [22].

Furthermore, oil-soaked ceramics and insulation in the EVALI patients' cartridges showed significant blackened and charred material, indicating that the liquid burnt at extremely high temperatures in the filaments. At high temperatures, metals and ceramics in the CVs catalyzed ketene formation from VEA responsible for EVALI [35, 38, 39]. It was observed that very high temperatures volatilized Ni, Cr, Zn, Cu, Pb, Au, and Sn in these devices [35]. Besides, devices with coil replacements more than twice a month had greater metal concentrations in the aerosol and vape liquids [12, 17].

Cannabis heating with volcano digit volatized the inherent terpenes and changed the elemental concentration of the BRM. In both low- and high-potency BRMs, Mg was the highest and Hg the lowest, while Sn was the same as Hg in high-potency samples. Due to the loss of water content and volatile terpenes with increased heating periods, concentrations of the “Big 4” metals such as Pb, As, Hg, and Cd increased for all BRM potencies, although below the standard inhalational levels. Besides Mg, Hg, Sn, Pb, As, and Cd, other metals in the raw cannabis were Al, Zn, Ba, Cu, Ni, Cr, V, Co, Mo, Li, and Sb. As there was no appreciable variation in concentrations between the various heat treatments, it was inferred that during the vaporization process, the metallic elements did not enter the cannabis vapor phase from the raw material; instead, they stayed in the samples [36]. However, in contrast to untreated and unvaped samples, higher temperatures and longer exposure times led to higher metal concentrations in cannabis oils [22].

#### 3.7.4. Design Characteristics of CV Devices

Changes in the liquid volume and product design may alter the physical characteristics of the active ingredients in the nicotine EC and CV aerosol. The CVs are ceramic cell cartridges with ceramic heating elements that contain connectors, a heating element, and additional surface area for producing aerosol from thicker cannabis liquids. The ceramic fiber wicking further insulates the cartridges from the liquid in CVs. However, in nicotine ECs, the silica wick absorbs the less viscous solvents, and the heating element in contact with the liquid produces more metals in the aerosol [23]. Accordingly, studies reported higher Cr, Ni, and Mn levels in the nicotine ECs and CV aerosol. The levels of Pb were similar, but higher levels of As, Ni, and Sn were present in nicotine ECs than in the CVs, which may be due to device component variations. The consumer use patterns also affect the metal levels in nicotine ECs and CVs [22]. When the vape device was used continuously, the heat produced by the first few puffs accelerated the metal leaching and produced a concentrated metal solution in the cartridge's latter sections [22]. Additionally, repeated heating and cooling of the coil caused the metal parts' expansion and contraction, leading to the metal particles' chipping from the damaged metal surfaces. The ceramic heating elements in the cylindrical chambers of CVs served as insulators and further increased the temperatures.

### 3.8. Health Implications of Metals Released From CVs

In the present review, more studies showed higher levels of Cu, Ni, Pb, Cr, Mn, Cd, Co, As, Hg, Zn, and Sn in the CV liquids, while the aerosols showed Cu, Ni, As, Hg, and Cr. In cartridges from EVALI response, patients' higher levels of Cu, Cr, Ni, and Pb were observed in CV liquid. In CV liquids from commercial cartridges, higher levels of metals beyond the usual regulated “Big 4” heavy metals, such as As, Cd, Hg, and Pb, were found [22]. Although evidence of heavy metals in CV liquids and aerosol is there, little information regarding their health implications is available. It is essential to consider the amount of metal breathed from these sources to assess their harmful consequences.

Metals like Pb, Cd, Hg, and Cr are carcinogenic. They are absorbed from the soil and exit the trichomes, which store cannabis oil and THC used by the consumers. The 2019 statewide outbreak of lung injuries and deaths of nicotine ECs and CVs users raised several concerns regarding their safety [40]. The Food and Drug Administration and the Centers for Disease Control concluded that illicitly produced cannabis concentrates containing VEA were the most likely cause of EVALI.

**Figure 4:**
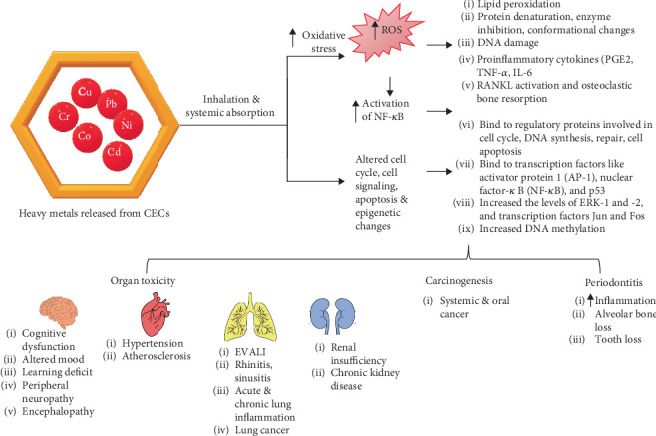
Health implications of metals released from cannabis vapes.

Pb and Cd were detected in the blood and urine of cannabis users [41]. Plausibly, low-quality fertilizers, irrigation water, or soils contaminated with Pb may increase their levels in the plant and may be released when the CVs are heated at high temperatures. Pb is limited to 0.5 *μ*g/g in inhalable marijuana products. It alters the oxidative, inflammatory, and immune-modulating pathways and causes neurological, pulmonary, urinary, and cardiovascular problems when it reaches its toxic levels (≤ 10 *μ*g/dL) [41, 42]. Pb interferes with vital intracellular events and activities by substituting calcium in tissues and metabolic pathways [43]. Besides Pb, nonoccupational exposure to Cr and Cd can occur from ECs [44]. Hexavalent Cr is carcinogenic and bioaccumulates in the body. At the same time, Cd accumulates in the liver, kidney, and other soft tissues and replaces the Zn (a divalent cation) in various enzymes and metalloproteins, leading to their malfunction. It plausibly substitutes other essential divalent cations like Cu [43]. It is likely that heavy metals like Pb, Cr, and Cd affect the body through increased ROS production, weakened antioxidant defense, negative impact on cell cycle, signaling pathways and apoptosis, enzyme inactivation, epigenetic effects, and multiple organ toxicity [44] (Figure 4).

Since not many studies specifically assess the harmful health effects of heavy metals produced from CVs, the next part examines how inhaling them affects oral and systemic health.

### 3.9. Systemic Health Implications of Various Metals Released From CVs

#### 3.9.1. Cardiovascular Risk

Exposure to toxic metals alters key biological pathways that regulate cardiac and vascular functions. These pathways include oxidative stress, lipid metabolism, myocardial electric perturbations, chronic inflammation, hypertension, kidney toxicity (primarily affecting the proximal tubule), impaired vascular endothelial function, cardiotoxicity, and epigenetic effects [43]. Pb causes inflammation and upsets the oxidant–antioxidant system's equilibrium, raising the risk of atherosclerosis and hypertension [42, 43, 45]. It raises blood levels of soluble adhesion molecules.

Modified contractility, disruption of regional blood flow, arterial stiffness, and consequent hypertension are some ways these alterations impact vascular functioning by affecting the movement of endothelial progenitor cells from bone marrow to peripheral circulation and blocking signaling processes triggered by vascular endothelial growth factor–receptor activation, and fine metal particles (particulate matter ≤ 2.5 microns) may even cause endothelial damage. Adhesion molecule expression in endothelial cells is increased by Cd, which also changes signaling, increases permeability, and causes inflammation and oxidative stress—all of which are proatherosclerotic stimuli [43]. According to studies, cellular exposure to Pb and Cd at varying doses enhanced the release of inflammatory mediators and proinflammatory cytokines, including prostaglandins, lipoxygenases, cyclooxygenase-2, and acute-phase proteins such as C-reactive protein [43]. Increased oxidative stress is the primary cause of these adverse effects. Pb and Cd compete with Cu and Zn, which are necessary for maintaining redox balance and cellular transport [43]. Divalent hazardous metals attach to sulfahydryl groups enhance mitochondrial and endoplasmic reticulum stressors and negate the antioxidant effects of glutathione, metallothionein, and Cu–Zn superoxide dismutase. Atherosclerotic plaque development is encouraged by elevated ROS levels because they raise the amount of oxidized lipids and lipoproteins. Systemic and cellular lipid metabolism are impacted by long-term exposure to environmental metals. Different circulating lipid profiles have been linked to the body's levels of Pb and Cd. Furthermore, various gene-environment interactions influence downstream transcription and gene expression through epigenetic effects such as DNA methylation and histone changes. By substituting the Zn needed for the action of DNA methyltransferases, histone acetyltransferases, histone deacetylases, and histone demethylases, Pb and Cd alter epigenetics. Cardiovascular disorders may result from long-lasting cardiac epigenetic changes brought on by Pb exposure [43].

#### 3.9.2. Neurological Diseases

Neurological effects of Pb and Cd are of most significant concern in children and adults as they are associated with reduced cognitive function at all ages. These heavy metals produce life-long decrements in cognition and neurological effects in infants and children. In children, Pb toxicity is mainly caused by its ingestion and absorption from the gastrointestinal tract. As CVs are very popular among teenagers and young adults, toxic Pb exposures may reduce their cognitive function, alter mood and behaviors, cause learning deficits, alter neuromotor and neurosensory function, peripheral neuropathy, and encephalopathy. Children between the ages of 6 and 16 exposed to Pb had lower attention, math, and reasoning scores [46]. In older people, decreased language, motor function, verbal and visual memory, and learning were linked to greater Pb concentrations in their tibia and patella [47]. Similarly, exposure to Cd was linked to poorer cognitive function, delayed recall, and poorer immediate learning [48]. Children's overall cognitive score was negatively impacted by prenatal exposure to volatile Cd from smoking moms [49]. Pb exposure increases the effects of proinflammatory cytokines in the brain, leading to neurotoxicity and CNS inflammation [50]. It disrupts the long-term potentiation necessary for information acquisition by hindering a noncompetitive, voltage-independent antagonist of the NMDA-r channel. Pb toxicity blocks neurotransmitter release and the NMDA receptor, leading to neurotoxicity and cognitive impairment [51]. Therefore, children and the elderly may develop harmful neurological effects from Pb and Cd exposure through CV aerosols.

#### 3.9.3. Respiratory Diseases and Lung Cancer

The metals, silica, and ceramic coils in nicotine ECs and CVs catalyzed ethenone formation from VEA at about 300°C triggered EVALI [38, 39]. Furthermore, metals like As, Ni, Cr, Cu, Pb, Sn, and Au in various CV device parts were associated with EVALI [33, 35]. Aerosol samples from EVALI patients' vape devices showed heavy metals released from the heating coil, which entered the lung epithelium through inhalation. CV aerosols are a mixture of vaporized gas-phase molecules and tiny oil droplets. At high temperatures, critical supersaturation of high molecular weight cannabinoids forms small particles inhaled along with the dissolved metals from the heating coil or the liquid and enter the systemic circulation [52].

A single puff of aerosol contained a high concentration of nanosized particles (20–300 nm) (4 million particles/cm^3^), while the vape liquid contained micron-sized particles [53]. These ultrafine particles can cause severe respiratory issues and damage the lungs, liver, kidneys, heart, and brain [12]. Lung inflammation, lung cancer, and other respiratory conditions like sinusitis and rhinitis are linked to Ni and Cr. Animal studies have shown that compared to micro Ni-oxide particles, nano Ni-oxide particles produced more widespread inflammatory lung lesions. Short-term exposure also caused acute lung inflammation, but long-term exposure led to lung fibrosis. Although vaping devices do not have a significant amount of metal, giant cell interstitial pneumonia was linked to Co in the CV liquid, which the patient had used [23, 54]. Cr and Cu cause respiratory irritation, chest pain, decreased lung function, and increased asthma risk. The DNA of the cultured cells was harmed by Co–Cr and Cr-oxide nanoparticles. In pregnant mice, Cu nanoparticles triggered pulmonary inflammation and immunomodulation in their offspring [55, 56]. Metals such as Al, Ca, Cu, Cr, Mg, Pb, Sn, and Zn in EC aerosols are hazardous and can cause lung cancer and respiratory tract infections. Lung cancer is mainly associated with excessive ROS production, genomic instability, apoptosis inhibition, and DNA damage [44, 57].

#### 3.9.4. Other Conditions

Pb can cause anemia by blocking ferrochelatase and ALAD, two essential heme biosynthesis enzymes [44]. It is both nephrotoxic and hepatotoxic. Cd has a detrimental effect on phosphorylation and inhibits protein phosphatases-1 [58]. These metals also interfere with the thyroid and steroid hormones and upset the endocrine system [59].

#### 3.9.5. Oral Health Implications of Various Metals Released From CVs

There is a dearth of literature on the oral health effects of metals released from the CVs. However, heavy metals like Pb, Cd, Ni, Cr, Co, and Cu are associated with the progression of periodontitis, oral cancer, and precancerous lesions [60, 61]. Their association is mainly due to the negative impact of these heavy metals on pathways of inflammation and DNA repair.

High concentrations of Cd were observed in the dental calculus of male smokers and betel quid chewers. Besides, the saliva also showed high levels of these metals in smokers [62, 63]. Oral mucosa is continuously stimulated by toxic heavy metals that penetrate the porous dental calculus from where they slowly leach into the oral cavity. The pathophysiology of oral squamous cell carcinoma may be influenced by the prolonged, continuous release of harmful trace heavy metals, which stimulates oral tissues such as the gingiva, inner mouth lining, and tongue border [62]. Carcinogenesis results from the replacement of Zn by Cd in Zn finger DNA binding domains and DNA repair enzymes. Likewise, elevated Cr was linked to oral squamous cell carcinoma because it has a role in angiogenesis, ROS formation, and DNA damage via several signaling pathways, including p53, NF-*κ*B, GADD45, Src kinase, and G proteins, all of which are important for cell division and proliferation [60]. Because of its capacity to enhance ROS generation and DNA damage, Co and Cu have also been linked to mouth cancer [60].

Likewise, Cd has several direct and indirect effects on periodontal bone levels. Cd is osteotoxic and indirectly affects bone metabolism by altering the parathyroid hormone levels [64]. It causes osteoblasts and osteoblast precursor cell degeneration in very low doses and increases osteoclast activity. By phosphorylating p38 MAPK, Cd activates p65 NF-*κ*B, which triggers IL-6 [65]. Through the RANKL cytokine pathway, IL-6 further increases osteoclast function, resulting in pathological alveolar bone resorption. As a result, Cd can damage alveolar bone and raise the risk of periodontitis.

Additionally, Pb exposure can increase TNF-*α* production, stimulating osteoclasts and worsening alveolar bone loss [66]. Besides, Pb and Cd exposure increases ROS generation, which may deplete the antioxidants and lead to oxidative stress. Pb causes lipid peroxidation and DNA damage by blocking antioxidant enzymes, including glutathione reductase and superoxide dismutase [67]. Cd also causes DNA mutations and mitochondrial dysfunction in periodontal tissues, which influence cellular death through the MAPK pathway [66]. Excessive ROS encourages periodontitis and alveolar bone resorption.

## 4. Strengths and Limitations

This review included studies to date focusing on heavy metals released from CVs, and it was observed that metals beyond the regulatory guidelines were released from them. However, these results cannot be generalized to all the CVs, as the studies were primarily experimental. Various research gaps were identified in the present review, including a lack of specific assessment of the harmful health effects of heavy metals produced from CVs in humans. Furthermore, a standard CV cartridge design and device characteristics like voltage settings, temperature, puffing profile, e-liquid composition, and flavorings were lacking in the studies, which caused the results to be noncomparable. As the federal government declared cannabis as illegal and only recently cartridges with CV liquids were legalized, there is scarce information on metal exposure, specifically from the nonpolar cannabis aerosols and their health implications. Future studies incorporating a standard exposure system for assessing the toxic dosage of heavy metals, the biomarkers affected, health effects through animal models and humans, and health implications of second-hand exposure are needed.

## 5. Conclusion

Metals like Pb, Co, Cr, Ni, and Cu are released from the structural components of CVs when operated at high temperatures. Factors like device age and storage, vape liquid composition and pH, operating temperature, and design characteristics of CVs influence metal dissolution. Heavy metal exposure from CVs may adversely affect various organ systems and produce respiratory, neurological, cardiovascular, and renal side effects. Owing to the increased popularity of the CVs among the young population, increased awareness of their harmful effects is imperative. Regulatory guidelines are needed to prevent CV liquid contamination from accessories or packaging. Furthermore, testing of metals beyond the “Big 4” should be advocated. Future research is warranted into the adverse health implications of heavy metals released from CVs through animal and human studies.

## Figures and Tables

**Table 1:** Evidence related to trace metals in cannabis vapes and their health implications.

**Author**	**Aims and objectives**	**Component of CV evaluated for metal detection**	**Metals in cannabis vape liquids**	**Metals in aerosol**	**Metals in CV components**	**Method for metal detection**	**Results and conclusion**
Muthumalage et al. [26]	Identified chemical constituents of counterfeit ECs and them to medical grade and CV cartridges	Vape liquid	Counterfeit cartridges: Cu, Ni, Pb, and Si (> 600 ppm)	—	—	ICP-MS	• Respiratory toxicants present in liquid and vapor phases of patient CVs and counterfeit cartridges not found in medical-grade CVs. • Inhaled hydrocarbons, oils, hazardous chemicals, and metals can result in lipoid pneumonia, which may trigger EVALI.

Wagner et al. [35]	Evaluated the internal components and make-up of the CVs related to 2019 EVALI patients and contrasted them with nicotine ECs from 2016 to 2019. Examined the components of polymers, metals, and ceramics subjected to heat in particular	CV device components	Ni, Cr, Cu, Pb, Sn, Au, and Si	—	Wire: Ni, Fe, Cr, Co, and S Battery contacts: Ni and Co Mouthpiece: Fe, Cr, and Ni	X-ray fluorescence SEM Fourier-transform infrared microspectroscopy	• CVs from EVALI patients had broken wire and burned ceramic heating components due to high temperatures. • More ceramic and polymer insulation in CV cartridges resulting in higher temperatures. • Combination of high temperatures, metals, and ceramics in CVs promotes VEA degradation to ketenes. • Optimum temperature settings may prevent decomposition of fluorinated microplastics and rubbers rich in Ni, Cr, Cu, Pb, Sn, Au, and Si, and detrimental effects might be mitigated.

Gonzalez-Jimenez et al. [23]	Created a method for analyzing the hydrophobic and hydrophilic aerosols of ECs for metals such as Al, Cr, Fe, Co, Ni, Cu, Cd, Sn, Ba, and Pb	Aerosol CV device components	—	Cu = 16.1 ng/10 puffs	Battery contacts: Ni, Cr, and Co	Triple quadrupole ICP-MS	• CV aerosols had metals but below the detection limit except for Cu. • Nicotine ECs showed presence of Pb and Sn.

Kubachka and Wilson [33]	Conducted elemental analysis of 65 EVALI-related cartridges	Vape liquid	Pb = 11.1* μ*g/g Ni = 477* μ*g/g Cu = 150* μ*g/g Zn = 120* μ*g/g Sn = 1.12* μ*g/g Cr = 3.89* μ*g/g Mn = 0.495* μ*g/g Cd = 0.033* μ*g/g Co = 0.968* μ*g/g Au = 0.93* μ*g/g	—	—	ICP-MS	• Metals are present in CV vape liquids.

Mallampati et al. [21]	Developed method for analysis of metals in CV aerosols from CV vape liquid and flower combustion	Vape liquid Aerosol Flower combustion	Before vaping: As = 7.33* μ*g/g Cd = 7.57* μ*g/g Co = 7.99* μ*g/g Cr = 8.02* μ*g/g Cu = 8.35* μ*g/g Hg = 3.44* μ*g/g Mn = 8.42* μ*g/g Ni = 8.01* μ*g/g Pb = 8.62* μ*g/g Sn = 9.85* μ*g/g After vaping: Increased concentration of As, Cd, Co, Cr, Cu, Hg, Mn, Ni, Pb, and Sn	Aerosol: As > Hg > Ni > Sn > Cu > Cr Flower combustion: As > Cd > Ni > Pb > Sn > Cu > Hg > Mn > Co > Pb	—	ICP-MS	• Following vaping, elevated concentrations of some metals in the concentrate suggest that the devices could be potential metal sources.

McDaniel et al. [22]	Analyzed CV components and aerosols for metals	Vape liquid Aerosol CV device components	Commercial cartridges Before vaping: Cr = 1.5* μ*g/g Cu = 6.4* μ*g/g Ni = 6.2* μ*g/g Mn = 0.82* μ*g/g Trace levels of big four metals: As (2.0 *μ*g/g), Cd (0.8 *μ*g/g), Hg (0.4 *μ*g/g), and Pb (1.2 *μ*g/g) After vaping: Increased levels of Cr, Cu, Ni, and Mn Model cartridges: Before heating: Cr = 0.15* μ*g/g Cu = 0.32* μ*g/g Hg = 0.25* μ*g/g Mn = 0.53* μ*g/g Ni = 0.089* μ*g/g 3 weeks (25°C) Cu = 2.9* μ*g/g Hg = 0.33* μ*g/g Mn = 0.63* μ*g/g Ni = 0.27* μ*g/g **7** months (25°C) Co = 0.12* μ*g/g Cr = 1.3* μ*g/g Cu = 88* μ*g/g Mn = 0.70* μ*g/g Ni = 41* μ*g/g Pb = 0.37* μ*g/g Sn = 0.6* μ*g/g 3 weeks (42°C) Cu = 0.39* μ*g/g Hg = 0.93* μ*g/g Mn = 0.43* μ*g/g Ni = 0.15* μ*g/g 7 months (42°C) Cd = 0.036* μ*g/g Cr = 1.5* μ*g/g Cu = 280* μ*g/g Mn = 0.84* μ*g/g Ni = 64* μ*g/g Pb = 13* μ*g/g Sn = 4.4* μ*g/g	Aerosol: In all groups: Cu, Ni, and Mn Terpenated oils (Cu = 0.04 mg/m^3^; Cr = 0.02 mg/m^3^; Ni = 0.05 mg/m^3^; Mn = 0.01 mg/m^3^) Unterpenated (Cu = 0.07 mg/m^3^; Cr = 0.12 mg/m^3^; Ni = 0.25 mg/m^3^; Mn = 0.02 mg/m^3^)	Heating coil and metal core: Ni, Cr, Cu, and Pb Mouthpiece–commercial cartridges: Cu, Mn, Ni, Pb, and Sn Model cartridges: Cr and Ni	ICP-MS	• Metals migrate into the cannabis oil and inhaled vapors and may result in greater inhalation of metals above the regulatory standards. • Direct combustion of cannabis flower and concentrate also show presence of metals. • Other metals than the normal As, Cd, Hg, and Pb should be included in the list of controlled metals because heated devices are a source of metal contamination.

Meehan-Atrash and Rahman [34]	Analyzed components of branded CVs	CV device components	Mg, Cr, Ni, Cu, Zn, Hg, and Pb	—	—	ICP-MS	• Inaccurate labeling *Δ*8-THC, cutting agents, and reaction products. • Heavy metals leached out of vaporizer components produce adverse health effects.

Gajdosechova et al. [12]	Investigated 12 different metal kinds in 20 legitimate and 21 illicit cannabis vape liquids	Vape liquid	Illegal samples: Pb = 50* μ*g/g Ni = 677* μ*g/g Zn = 426* μ*g/g Legal samples: Cu = 485* μ*g/g Other metals: Co, Cr, Mn, V, and Na	—	—	SEM Laser ablation ICP-MS	• The metal percentage of legal cannabis vape liquids varied among vape liquids made from the same cannabis lot. • Metal particles found in the vape liquid of unused cannabis devices.

Wang et al. [36]	Examined how, during the vaporization process, metallic components from cannabis material were transported to cannabis vapor	Cannabis plant material	—	Mg, Al, Zn, Ba, Cu, Ni, Cr, V, Pb, Co, Mo, Li, As, Cd, Sb, and Hg	—	ICP-MS	• Mg, Al, Zn, Ba, Cu, Ni, Cr, V, Pb, Co, Mo, Li, As, Cd, Sb, and Hg in all types of cannabis material. • Mg present in highest concentration. • No elemental contaminants moved from the raw material into the vapors during the vaporization process, since the metallic elements persisted in the samples even after varying heat treatments (no heat, 30 s heat, 70 s heat, and 70 s heat with air). • Elemental contaminants are found in the cannabis vapors when the material is heated to a significantly higher temperature for a long time.

## Data Availability

Data sharing is not applicable to this article as no new data were created or analyzed in this study.
